# Supplementary material for: Thermal Evaporation Synthesis of Vertically Aligned Zn2SnO4/ZnO Radial Heterostructured Nanowires Array
Source: Nanomaterials (Basel). 2021 Jun 6;11(6):1500. doi: 10.3390/nano11061500 (PMC8227187; doi:10.3390/nano11061500)
Supplement: Supplementary file 1 [file nanomaterials-11-01500-s001.zip › nanomaterials-1213918-SI.pdf]

Supplementary Material for

Thermal Evaporation Synthesis of Vertically Aligned Zn_2SnO_4/ZnO Radial Heterostructured Nanowire Arrays

Gill Sang Han,[1,a] Min Jae Kang, [2,3,a] Yoo Jae Jeong,[2,3] Sangwook Lee,[4,*] In Sun Cho[2,3,*]

[1]School of Advanced Materials Science and Engineering, Sungkyunkwan University, Suwon 16419, South Korea

[2]Department of Materials Science and Engineering, Ajou University, Suwon 16509, South Korea

[3]Department of Energy Systems Research, Ajou University, Suwon 16509, South Korea

[4]School of Materials Science and Engineering, Kyungpook National University, Daegu 41566, South Korea

[a]These authors contributed equally.

* Corresponding authors:

Sangwook Lee

Email: wook2@knu.ac.kr

In Sun Cho

Tel: +82-31-219-2468; Fax: +82-31-219-1613; Email: insuncho@ajou.ac.kr

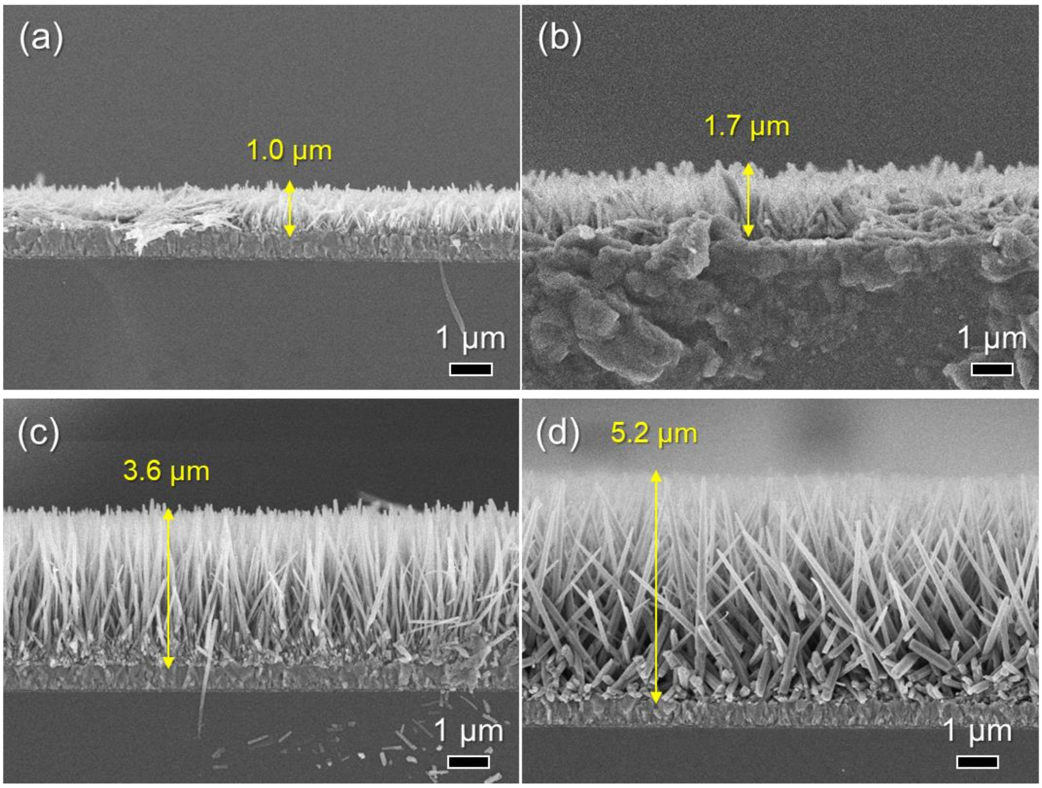

Figure S1. Effect of NH_4OH amount on the morphology and length of ZnO NWs synthesized at 100 °C/2h with polyethyleneimine (PEI, 1 g) and addition of NH_4OH (a) 1 ml, (b) 2 ml, (c) 3 ml, and (c) 4 ml.

The addition of NH_4OH affected the concentration of Zn-complex that largely affects the Zn solubility in the growth solution. Consequently, the supersaturation, i.e., nuclei density, can be controlled. Additionally, the pH affected growth rate [Nanoscale Research Letters 13 (2018) 249; Inorg. Chem. 45 (2006) 7535–7543]. Therefore, the aspect ratio and density of ZnO NWs increased with the addition of NH_4OH.

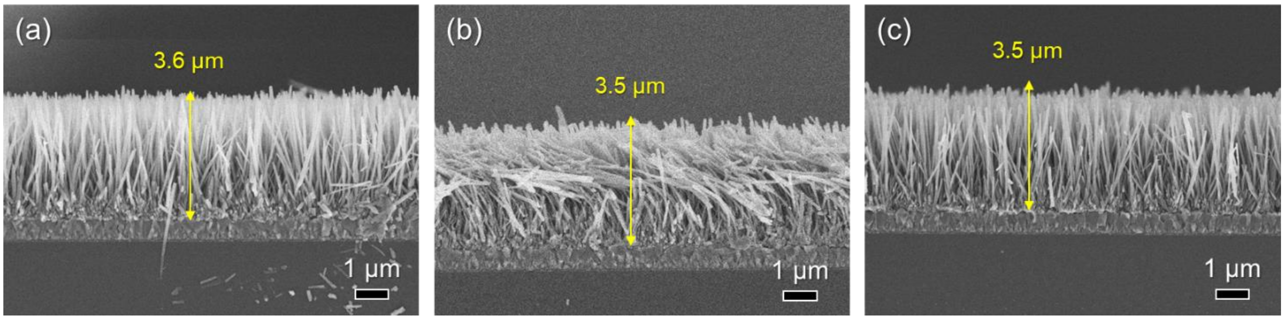

Figure S2. Effect of growth time on the morphology and length of ZnO NWs synthesized at 100 ℃ with polyethyleneimine (PEI, 1 g) and addition of NH_4OH (3 ml). (a) 2 h. (b) 4 h. (c) 6 h.

The growth time has little impact on the morphology and length of ZnO NWs. This result indicates that the growth rate is fast and whole growth occurs within 2 h. After 2 h growth, most of the precursors are consumed, i.e., no additional growth occurred.

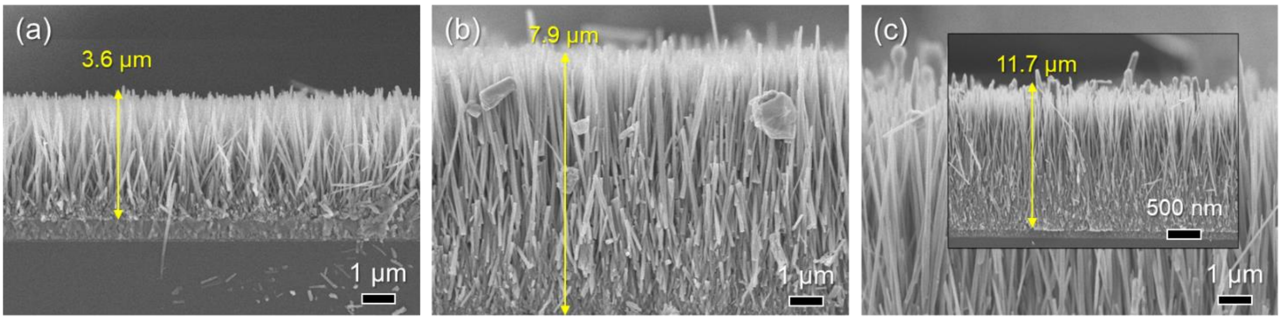

Figure S3. Effect of growth cycle on the morphology and length of ZnO NWs synthesized at 100 °C/2 h with polyethyleneimine (PEI, 1 g) and addition of NH_4OH (3 ml). (a) 1 cycle. (b) 3 cycles. (c) 5 cycles.

As repeating the growth cycles, the nanowire length increases linearly from 3.6 μm to 11.7 μm. There was no branch growth. However, NW density at the bottom increased.

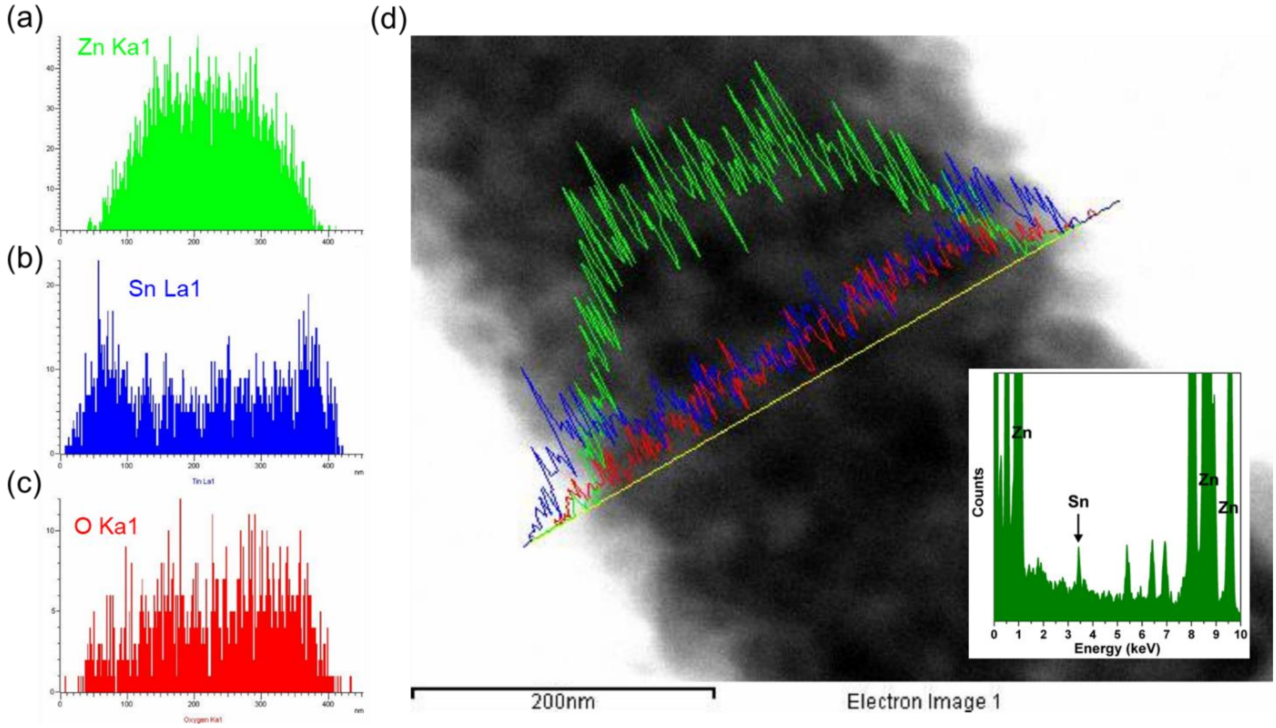

Figure S4. TEM-EDS analysis of ZSO/ZnO NW. (a-c) EDS line scan spectra. (d) STEM image. Inset shows EDS spectrum.

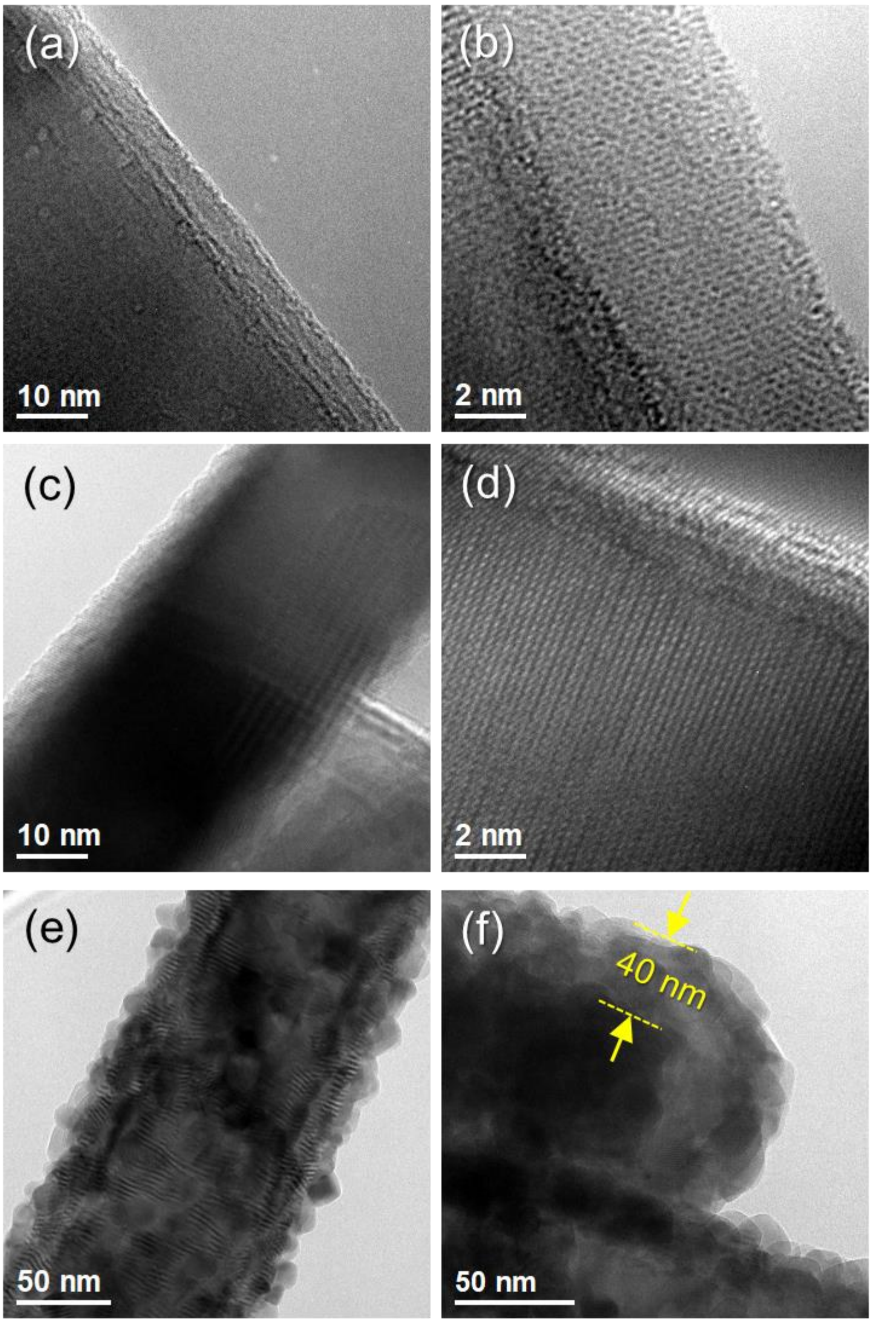

Figure S5. TEM and HR-TEM images of ZSO/ZnO NWs. (a,b) Zn/Sn evaporation for 30 min, without post-annealing. (c,d) Zn/Sn evaporation for 30 min, with a post-annealing at 550 °C/1h. (e,f) Zn/Sn evaporation for 2 h, with a post-annealing at 550°C/1h.

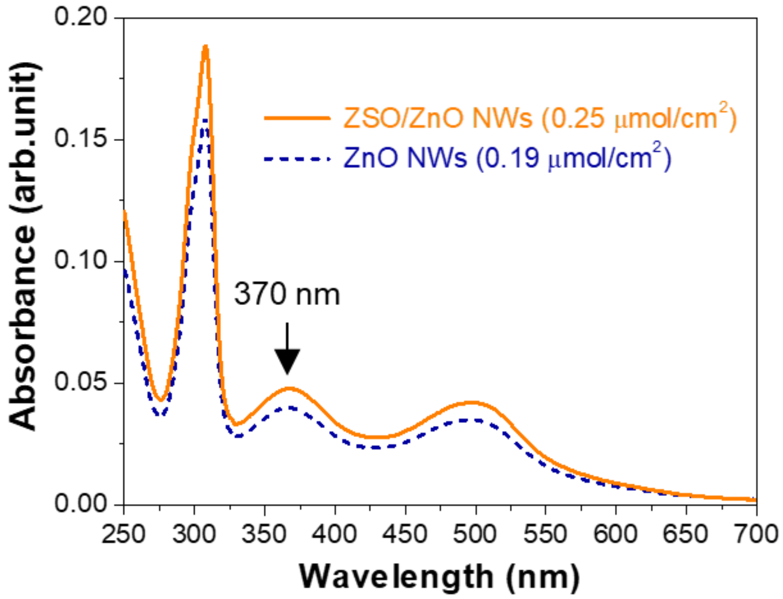

Figure S6. Amount of dye adsorption (N719) measurement by UV-Vis spectroscopy. The amount of dye adsorption value was obtained by comparison of the peak intensity at 370 nm with that of the standard solution. As shown in the parenthesis, the ZSO/ZnO HNA exhibited 130% larger dye adsorption (0.25 μmol/cm^2), indicating a larger surface (or surface roughness) area than the ZnO NW (0.19 μmol/cm^2).
